# The Effect of Axial Compression and Distraction on Cervical Facet Cartilage Apposition During Shear and Bending Motions

**DOI:** 10.1007/s10439-022-02940-1

**Published:** 2022-03-07

**Authors:** Ryan D. Quarrington, Darcy W. Thompson-Bagshaw, Claire F. Jones

**Affiliations:** 1 Adelaide Spinal Research Group, Centre for Orthopaedic & Trauma Research, Adelaide Medical School, The University of Adelaide, Level 7, Adelaide Health and Medical Sciences Building, North Terrace, Adelaide, SA 5000 Australia; 2 School of Mechanical Engineering, The University of Adelaide, Level 7, Adelaide Health and Medical Sciences Building, North Terrace, Adelaide, SA 5000 Australia

**Keywords:** Cervical spine, Computer model, Facet joint apposition

## Abstract

During cervical spine trauma, complex intervertebral motions can cause a reduction in facet joint cartilage apposition area (CAA), leading to cervical facet dislocation (CFD). Intervertebral compression and distraction likely alter the magnitude and location of CAA, and may influence the risk of facet fracture. The aim of this study was to investigate facet joint CAA resulting from intervertebral distraction (2.5 mm) or compression (50, 300 N) superimposed on shear and bending motions. Intervertebral and facet joint kinematics were applied to multi rigid-body kinematic models of twelve C6/C7 motion segments (70 ± 13 year, nine male) with specimen-specific cartilage profiles. CAA was qualitatively and quantitatively compared between distraction and compression conditions for each motion; linear mixed-effects models (*α* = 0.05) were applied. Distraction significantly decreased CAA throughout all motions, compared to the compressed conditions (*p* < 0.001), and shifted the apposition region towards the facet tip. These observations were consistent bilaterally for both asymmetric and symmetric motions. The results indicate that axial neck loads, which are altered by muscle activation and head loading, influences facet apposition. Investigating CAA in longer cervical spine segments subjected to quasistatic or dynamic loading may provide insight into dislocation and fracture mechanisms.

**Supplementary Information:**

The online version contains supplementary material available at 10.1007/s10439-022-02940-1.

## Introduction

The bilateral facet joints of the subaxial cervical spine play an important role in load-bearing and kinematics of the neck. They bear over 64% of axial load in the cervical spine^20,22^ and are responsible for coupled intervertebral motions in axial rotation and lateral bending.^4,19^ The facets also protect the spinal cord by preventing excessive intervertebral axial rotation, lateral bending, and anterior shear motions.^4^ Evidence of facet joint subluxation (translation of one facet relative to the other) is often identified on medical images following cervical spine trauma,^1,29,34^ but facet articulation during complex cervical intervertebral motions has not be defined with respect to apposition area and location.

Severe cases of facet subluxation can result in cervical facet dislocation (CFD), where the superior facet of the joint translates anteriorly (relative to the inferior facet) such that the articular surfaces are no longer in contact, producing a ‘perched’ or ‘locked’ facet. If concomitant fracture occurs, the region(s) of articular apposition likely dictate the fracture type and location.^26^ Facet joint apposition area and region are dictated by bone and cartilage geometry,^30,36^ posterior element deflection,^26,30^ and the *local* instantaneous intervertebral loads and motions. Muscle forces impose intervertebral *compressive* loads^18^ that may restrict flexion and inter-facet separation, hence maintaining or increasing facet apposition during trauma. In contrast, superimposed intervertebral *distraction*, such as that observed during inertially-produced CFD,^11,21^ likely reduces facet joint apposition, which may reduce the likelihood of concomitant posterior element fracture. The effect of axial compression, versus distraction, on the magnitude and region of facet joint apposition during intervertebral motion has not previously been reported.

Existing experimental methods for measuring *ex vivo* facet joint apposition area alter the joint structures and kinematics,^12,13,23,33,36,39^ and typically overestimate true apposition area,^2,14^ but computational models of experimental kinematics data can provide a method of estimating joint apposition without compromising the joint. Kinematic data, combined with specimen-specific geometries of spine segments, has been used to estimate facet cartilage apposition area in the lumbar^7^ and cervical spine^30^ Unlike finite element (FE) modelling, where external forces are applied to a model to predict the propagation of stress and strain in the tissue(s), kinematic modelling applies measured kinematic data to the modelled (rigid) anatomic geometry to derive geometric relationships (such as facet joint articulation area) that cannot be measured directly.

The aim of this study was to estimate bilateral facet apposition area throughout intervertebral flexion (10°), anterior shear (1 mm), axial rotation (4°), and lateral bending (5°) motions, using specimen-specific, multi rigid-body kinematic C6/C7 computational models. The effect of superimposed intervertebral axial distraction and compression on facet apposition estimates at the rotation/displacement limit of each motion was compared.

## Methods

### Experimental Data

Twelve subaxial cervical spine motion segments (C5-T1 or C6/C7) were dissected from fresh frozen human cadavers (mean donor age 70 ± 13 years, range 46–88; nine male) while maintaining the C6/C7 intervertebral joints and discoligamentous tissues; the C6/C7 level is most often dislocated (± facet fracture) during cervical spine trauma.^29^ Fiducial markers (*N *= 6 per specimen, 2 mm diameter aluminium spheres) were embedded in the C6 and C7 vertebra, and computed tomography scans (CT; SOMATOM Force, Siemens, Erlangen, Germany; 0.23 × 0.23 × 0.4 mm voxel size) were obtained to generate three-dimensional (3D) specimen-specific, three rigid-body, hard tissue models of the C6 and C7 vertebrae, as previously described.^30^ Custom, light-weight, motion capture marker carriers were attached to the C6 and C7 vertebral bodies with K-wires, and fixed bilaterally to the inferolateral corners of the C6 inferior facets using cyanoacrylate adhesive (Loctite 401, Henkel, Düsseldorf, Germany) (Figure S1 in Supplementary Material).

Each specimen underwent mechanical testing, as previously described.^27^ Briefly, the distal thirds of C6 and C7 (plus C5 and T1 for the four-vertebra specimens) were embedded in molds using polymethylmethacrylate (Vertex Dental, Utrecht, Netherlands) to fix the specimen within the test space of a six-axis materials testing machine (8802, Instron, High Wycombe, UK) (Figure S1). Three cycles of non-destructive anterior shear (1 mm, 0.1 mm/s), flexion (10°, 1 °/s), right axial rotation (4°, 1°/s) and left lateral bending (5°, 1°/s) were applied. Each motion was superimposed with each of three axial loading conditions: a ‘neutral’ condition, replicating head-weight loading by applying a 50 N axial compression force;^3,9^ a ‘compressed’ condition, where the 300 N compression force simulated the loading experienced due to neck muscle bracing;^3,5,8,10,16,25^ and, a ‘distracted’ condition to simulate intervertebral separation due to inertial head loading,^11,21^ in which 2.5 mm of intervertebral distraction corresponded to the largest non-destructive axial separation previously reported for *ex vivo* cervical motion segments.^32^ The displacement/rotation limits were based on *in-vivo* ranges of motion,^15,24,31,38^ and a two-second position “hold” was applied at the peak of each displacement/ rotation and between each motion. The order of application of the axial conditions and the motions were block randomized for each specimen, amounting to a total of 12 tests per specimen. Forces and moments were monitored using a six-axis load cell (MC3A-6-1000 ± 4.4 kN, AMTI, Massachusetts, USA). Specimen hydration was maintained using saline-soaked gauze and saline spray.

Prior to testing, each C6 and C7 fiducial marker, and anatomical landmarks on the C6 inferior facets, were digitized using a four-marker wand (Optotrak Certus, Northern Digital Inc., Ontario, Canada) while the specimen was ‘unloaded’ (< 10 N axial compression, all other loads/moments ~0 N or Nm). Motion capture data were acquired at 200 Hz (Optotrak Certus, Northern Digital Inc., Ontario, Canada; system bias < 0.09°, and precision = 0.006°^28^). Actuator positions were collected at 600 Hz using a data acquisition system (PXIe-1073, BNC-2120 & PXIe-4331 (x2), National Instruments, USA).

Experimental data were filtered and processed using custom MATLAB code (R2020a, The Mathworks, Massachusetts, USA), and 6 degree-of-freedom load profiles for each specimen have been published.^27^ Second-order, two-way, Butterworth low-pass filters were used for loads and actuator positions (cut-off frequency = 100 Hz), and for motion capture data (cut-off frequency = 30 Hz).

### Specimen-Specific Computer Models

Three-dimensional hard-tissue models of C6 and C7 were generated from the high-resolution CT scans of each specimen using 3D analysis software (Amira 6.4.0, Thermo Fisher Scientific, Massachusetts, USA). Specimen- and facet-specific, spatially-varying thickness cartilage profiles were generated by extruding a thickness mapping function from each osteochondral facet surface.^37^ The maximum thickness of these profiles were estimated from facet joint space measurements performed on high-resolution sagittal CT slices.^30^

**Figure 1:**
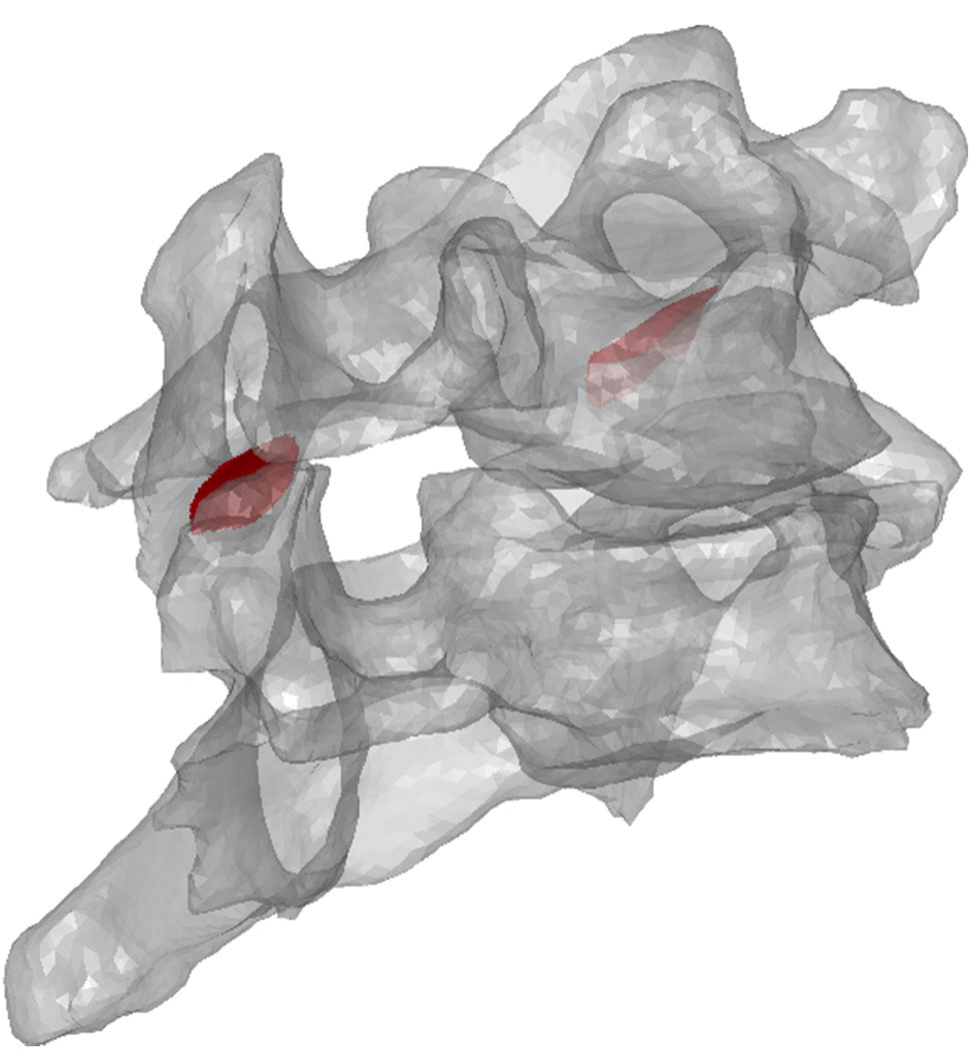
Antero-lateral view of the computer model of H009 in 10° of flexion, with 50 N of superimposed compression. The regions of facet joint articulation, projected onto the C6 articular surfaces, are indicated in red.

Custom MATLAB code transformed the models from the CT coordinate system to the experimental (motion capture) coordinate system by co-registering the locations of the fiducial markers, and the motion-capture data was applied to the models to recreate the experiments. To account for deflections of the C6 posterior elements (including the bilateral facets), relative to the vertebral body,^26,27^ the C6 vertebra was considered as three separate rigid-bodies: the vertebral body, and the left and right inferior facets. Due to the anatomy of the facet joints (and surrounding structures), attaching marker carriers to the C7 facets was not possible; hence, the C7 vertebrae was modelled as a single rigid-body. The three *vertebral body fiducial marker locations* at each test frame were used to determine the C6 and C7 vertebral body motions, and the C6 inferior facet landmarks positions were used to account for posterior element deflections. Facet joint cartilage apposition area (CAA) at each test frame was estimated by summing the area of intersecting (elements crossing) and penetrating (elements from one body lying internal to the volume of the second body) opposing cartilage mesh elements, as previously described.^30^ Regions of apposition and the corresponding centroids were projected onto the C6 facet articular surface of each joint for qualitative comparison between motions and axial conditions (Fig. 1).

### Statistics

Peak CAA for each facet was calculated at the displacement/rotation limit of the third cycle, for each axial condition. Statistical analyses were performed using SPSS v26 (IBM, Illinois, USA). For each motion, a linear mixed-effects model (LMM) was developed to identify if axial condition was significantly associated with CAA (*α* = 0.05). Each model was adjusted for facet side (left/right), and a random effect of facet side nested within donor identifier was included.

## Results

**Figure 2:**
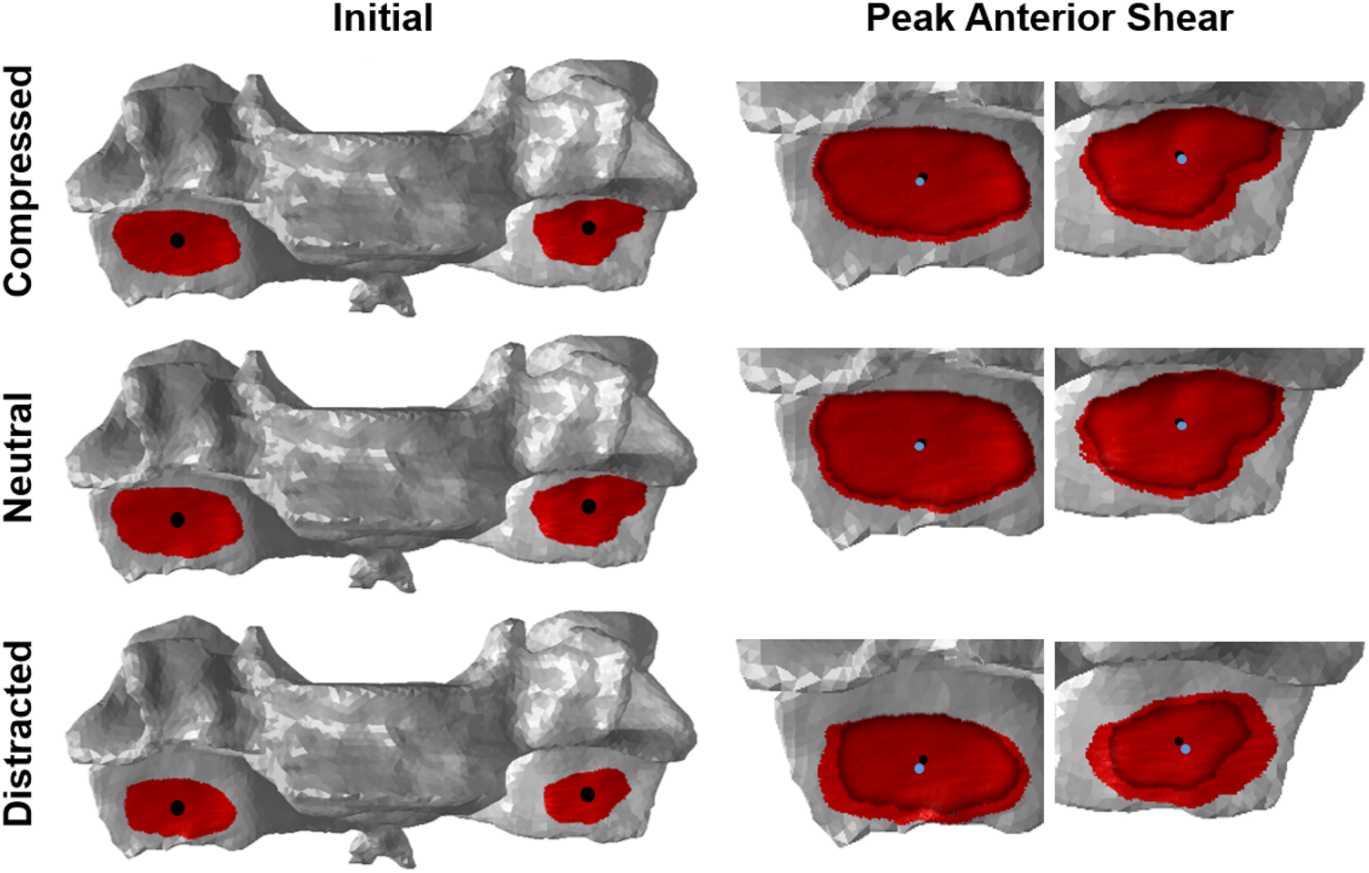
Anterior view of the computer model of H009, C6 vertebrae. Apposition regions at the beginning of each test and at peak anterior shear motion (columns), for each axial condition (rows), are indicated by the red shading on the articular surfaces of the inferior facets. In the right column, the articular surfaces are cropped and enlarged to demonstrate the shift in the apposition area centroid location from the beginning (black dot and outline) to the anterior shear motion limit (blue dot).

Rigid-body computer models were generated for eleven of the twelve cervical spine motion segments (see video in supplementary material); one specimen (H004) was excluded from all analyses due to pathological joint morphology. Visualisation of the model demonstrated that at the limit of each motion, superimposed distraction caused apposition to be concentrated near the facet tip, while the compressed condition resulted in cartilage apposition closer to the C6 pedicles (Fig. 2 left column).

**Figure 3:**
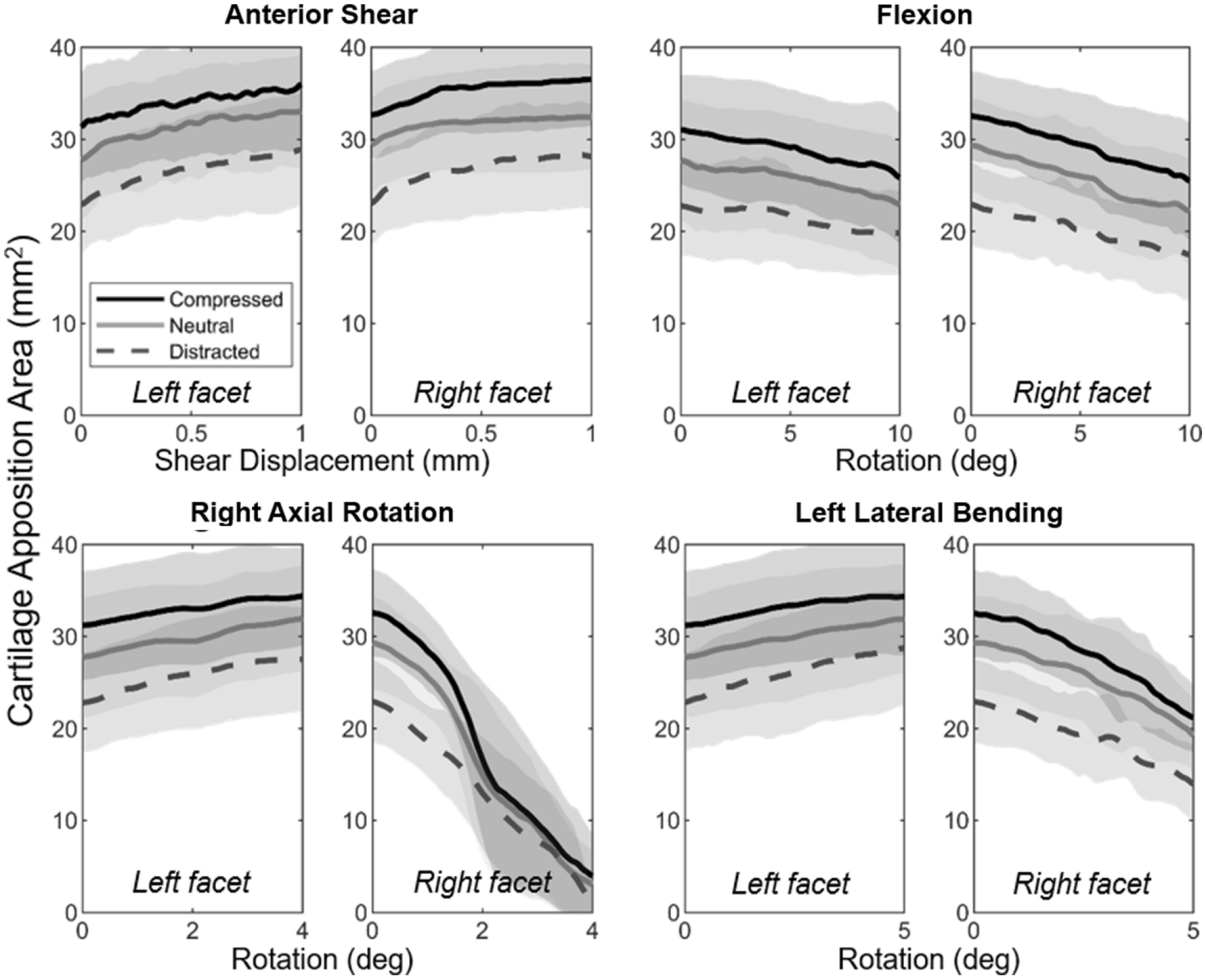
Mean (± S.D.) cartilage apposition area versus displacement/rotation for the loading region of each motion, in each axial condition.

The initial CAA (prior to motion) was larger for tests with superimposed compression (‘compressed’ and ‘neutral’ groups) than for the distracted group (Fig. 3; compressed: 31.90 ± 1.62 mm^2^, neutral: 28.57 ± 1.75 mm^2^, distracted: 22.91 ± 1.50 mm^2^). An increase in anterior shear displacement resulted in an increase in CAA for both facets, for all axial conditions, while flexion rotation symmetrically reduced CAA. During right axial rotation and left lateral bending, CAA increased for the left facet and decreased for the right facet for all axial conditions.

**Figure 4:**
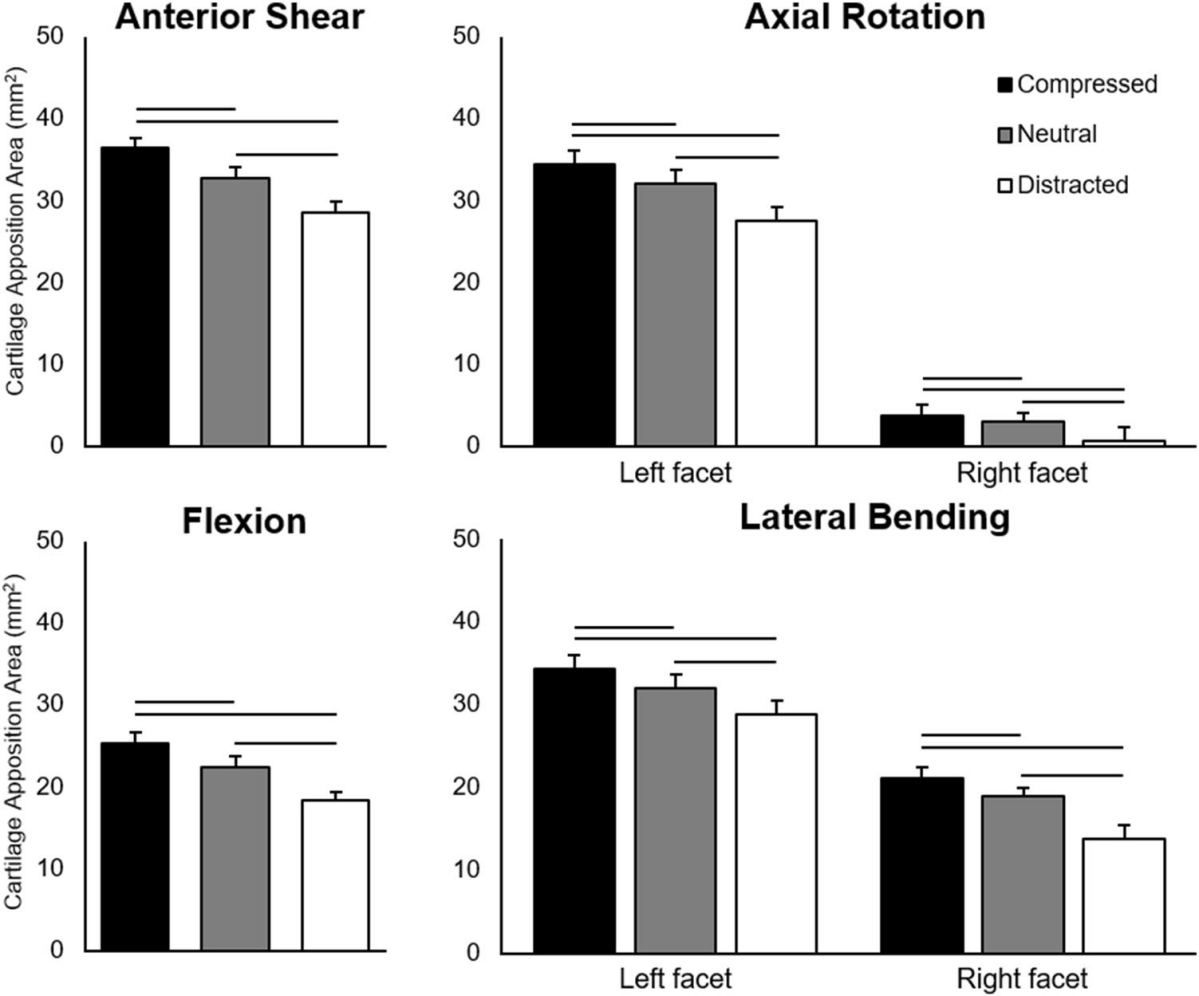
Mean (+ S.E.M.) cartilage articulation area for compressed, neutral, and distracted axial conditions at each motion limit. Left and right facet measurements are grouped for those outcomes with no significant difference between sides. Significant difference between axial conditions for each motion, as determined by Bonferroni-adjusted post-hoc analysis of the final multivariable LMMs (*α* = 0.05), are indicated.

At the limit of each motion, CAA was significantly larger when superimposed with compression, compared to the neutral and distracted conditions, and the neutral axial condition significantly increased CAA at peak when compared to distraction (*p *< 0.001 for all; Fig. 4, Table 1). The aforementioned asymmetry in CAA for the axial rotation and lateral bending tests was also statistically significant at peak motion (*p *< 0.001 for both; Fig. 4, Table 1).

**Table 1:** Final multivariable linear mixed-effects models for cartilage apposition area (mm^2^), for each motion.

Variable	EMMs (95% CI)	*p *Value	Estimate (95% CI)
*Anterior shear*			
Axial state		**< 0.001**	
Compressed	36.4 (33.8, 39.0)	**< 0.001**	3.6 (2.4, 4.8)
Distracted	28.6 (26.0, 31.2)	**< 0.001**	− 4.2 (− 5.4, − 3.0)
Neutral*	32.8 (30.2, 35.4)	–	–
Facet side		0.874	
*Axial rotation*			
Axial state		**< 0.001**	
Compressed	19.1 (17.1, 21.2)	**< 0.001**	1.6 (0.1, 3.1)
Distracted	14.1 (12.0, 16.1)	**< 0.001**	− 3.4 (− 4.9, − 1.9)
Neutral*	17.5 (15.4, 19.5)	–	–
Facet side		**< 0.001**	
Right	2.5 (-0.3, 5.2)	**< 0.001**	− 28.9 (− 32.7, − 25.0)
Left*	31.3 (28.6, 34.1)	–	–
*Flexion*			
Axial state		**< 0.001**	
Compressed	25.3 (22.6, 27.9)	**< 0.001**	2.9 (1.4, 4.3)
Distracted	18.3 (15.7, 21.0)	**< 0.001**	− 4.0 (− 5.5, − 2.6)
Neutral*	22.4 (19.7, 25.0)	–	–
Facet side		0.670	
*Lateral bending*			
Axial state		**< 0.001**	
Compressed	27.7 (25.4, 30.0)	**< 0.001**	2.3 (1.0, 3.6)
Distracted	21.2 (18.9, 23.6)	**< 0.001**	− 4.2 (− 5.5, − 2.9)
Neutral*	25.4 (23.1, 27.7)	–	–
Facet side		**< 0.001**	
Right	17.9 (14.8, 21.1)	**< 0.001**	− 13.8 (− 18.2, − 9.3)
Left*	31.7 (28.5, 34.8)	–	–

Bold indicates a statistically signficiant association between the variable and the outcome measure (*p* < 0.05)

*Indicates reference category. Sub-category *p *values were determined from post-hoc comparison of estimated marginal means (EMMs), using Bonferonni correction for multiple comparisons

## Discussion

Despite bearing a substantial portion of overall neck loading, and contributing to head and neck kinematics, there has been little research into changes in the magnitude and region of facet joint apposition during intervertebral motion in the lower cervical spine. During cervical spine trauma, a reduction in facet joint apposition area likely increases the risk of CFD, while the anatomical region of contact (or lack thereof) dictates the presence and type of concomitant posterior element fractures. This study used computational reconstructions of experimental data to show that superimposed intervertebral compression significantly increased apposition area and moved the region of contact towards the pedicles, whereas superimposed distraction caused the smaller area of apposition to be closer to the facet tip. These observations were consistent for all facet joints and motions, despite asymmetric motions eliciting an asymmetric facet response.

Facet joint CAA estimates in the current study were within the range of those reported in the literature. Womack et al.^36^ placed Tekscan pressure mapping sensors within the C4–C6 facet joints of six cadaver cervical spines and applied pure moments up to ± 2 Nm in each rotational direction, with 40 N of axial compression superimposed. Facet joint apposition area estimates for the neutral axial condition (50 N compression) in the current study were comparable at equivalent displacements,flexion (10°; mean CAA ± S.D. = 22.4 ± 6.3 mm^2^ vs Womack: 26.8 ± 23.6 mm^2^), lateral bending (5°; 31.9 ± 5.9 vs Womack: 45.1 ± 22.7 mm^2^), and axial rotation (4°; 32.0 ± 5.6 vs Womack: 36.1 ± 24.7 mm^2^). The greater variation in apposition area amongst specimens measured using the pressure films could be, in part, due to altered joint kinematics caused by the joint capsule compromise required to insert them,^39^ while the larger mean values are consistent with observations that Tekscan pressure sensors tend to overestimate joint contact areas.^2^

The results of the current study are consistent with previous reports that the cervical facet joints are *unloaded* (corresponding to a decrease in apposition area) during intervertebral flexion,^6,12^ and that they are further unloaded when distraction is superimposed on intervertebral flexion.^27^ This observation seems to support the long-held belief that CFD is a *distractive-flexion injury,*^1^ where supraphysiologic flexion with superimposed distraction reduces facet apposition such that dislocation can occur. However, this motion alone is unable to produce CFD in cadaver head-neck specimens,^17^ so further investigation into the spinal kinematics leading to CFD is required to understand how these injuries occur. In contrast to distraction, superimposed intervertebral compression led to significantly larger CAA at the limit of each motion. This is consistent with observations from our group’s previous experimental work, in which increased axial compression was associated with larger facet surface strains and deflections.^27,28^

During each motion, increasing amounts of intervertebral separation shifted the region of joint contact towards the facet tip, while compression caused contact to occur more proximally (i.e. closer to the pedicles) (Fig. 2). This is an intuitive result given the inclined sagittal-plane orientation of the facet joints in the lower cervical spine, but (to the author’s knowledge) this is the first time that it has been demonstrated with experimental data. The shift in contact location may provide insight into the mechanisms underlying facet fractures in the cervical spine. Mechanical testing of the bilateral facets of isolated subaxial cervical vertebrae has demonstrated that when the region of facet contact translates distally along the facet articular surface, fracture occurs through the facet tip; and, in tests in which the contact point remains in the centre of the facet, fracture occurs through the pedicles or articular pillars.^26^ Taken together, these results suggest that if the *local* force vector causes increased intervertebral separation (i.e. has a component causing intervertebral distraction and/or flexion) then fracture through the facet tip, or CFD without fracture, may occur, whereas a force vector with a sufficient component that causes compression is more likely to cause a ‘shearing’ injury with fracture of the articular pillar or pedicles.

Regarding spinal trauma, inference based on the results of the current study is limited by aspects of the mechanical testing, as previously described.^27^ Briefly, the loading rates applied are lower than those expected during cervical spine injury,^35^ but quasi-static motions were necessary to accurately characterize the mechanical response of the cervical facets using a motion-capture system. Furthermore, the applied motions were fully constrained with a fixed centre of rotation, which likely only represents a subset of real-life trauma, but quantitative information about changes in facet joint apposition during constrained intervertebral motions can assist with the development of improved neck injury criteria; this data may also be used to validate the outputs of advanced FE and other computational models of the cervical spine, where the well-defined, constrained C6/C7 intervertebral motions can be applied as inputs. Although these experiments were non-destructive, extrapolation of the CAA results can provide valuable insight into the likely mechanisms of cervical facet trauma. Future studies could verify these inferences by modelling supraphysiologic intervertebral motions. Finally, changing from C5-T1 to C6/C7 specimens was necessary, but no statistically significant difference in the mechanical behavior of the two specimen types was observed.^27^

The limitations associated with the modelling method used in this study have been discussed in detail.^30^ Briefly, spatially-varying thickness cartilage profiles, which included joint-specific maximum thickness estimates, were applied to the osseous articular facet surfaces, but the accuracy of these profiles could not be directly validated because the cartilage could not be accurately visualised on medical imaging. Cartilage deformation, which would likely have increased apposition area estimates, was not modelled and incorporating C7 posterior element deflections (if logistically possible) would produce more accurate CAA values. However, the CAA produced using the current method were comparable to those measured experimentally and estimated by validated computer models,^36^ and repeated measures analyses ensured that these limitations did not confound the observed association between axial condition and CAA.

The current study provides C6/C7 facet joint apposition area data during constrained intervertebral shear and bending motions, with three superimposed axial loading conditions. The results demonstrate that, for all motions, increased intervertebral distraction significantly decreased CAA, when compared to the compressed conditions, and the articulating region shifted towards the facet tip. This supports the theory that global head-neck loading that causes intervertebral separation (such as that which might occur during inertial head loading) increases the risk of facet tip fracture, or CFD without fracture, while superimposed compression (due to neck muscle activation, for example) is likely associated with more severe fractures of the posterior elements. To further investigate this, the modelling approach of the current study could be applied to experimental kinematics data from cervical spinal segments exposed to supraphysiologic or traumatic intervertebral motions. The information reported contributes to advancing our understanding of the biomechanics underlying neck trauma, which may assist with developing improved injury criteria and prevention devices.

## Supplementary Information

Below is the link to the electronic supplementary material.

Supplementary file1 (PDF 402 kb)

## Abbreviations

CFD: Cervical facet dislocation
CAA: Cartilage apposition area
