# Supplementary material for: The Effect of Axial Compression and Distraction on Cervical Facet Cartilage Apposition During Shear and Bending Motions
Source: Ann Biomed Eng. 2022 Mar 7;50(5):540–8. doi: 10.1007/s10439-022-02940-1 (PMC9001226; doi:10.1007/s10439-022-02940-1)
Supplement: Supplementary file 1 — Supplementary file1 (PDF 402 kb) [file 10439_2022_2940_MOESM1_ESM.pdf]

SUPPLEMENTARY MATERIAL

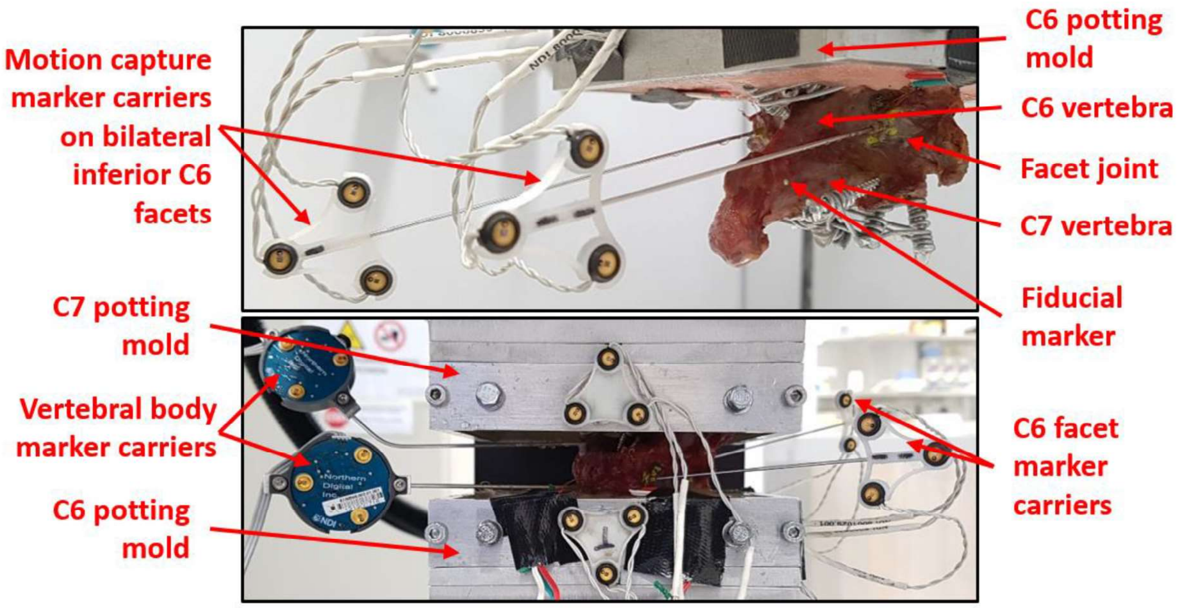

Figure S1: Specimens were embedded into potting molds containing polymethylmethacrylate in two stages. Firstly, the superior level(s), including the distal third of C6 and screw/wire constructs to assist with fixation, were embedded (top panel). Prior to embedding the inferior anatomy, Optotrak motion capture marker carriers were fixed to the C6 inferior facet tips, bilaterally. The embedded and instrumented specimen was then fixed into the test space of an Instron materials testing machine, and additional marker carriers were fixed to the C6 and C7 vertebral bodies via Kirschner wires (bottom panel).
